# Institutional dynamics, stakeholder interactions, and sustainability pathways in North Toraja's Arabica coffee sector: a systems approach to inclusive agribusiness transformation

**DOI:** 10.3389/fsoc.2026.1684951

**Published:** 2026-03-12

**Authors:** Putra Astaman, Rahim Darma, Riad Azkar, Nitty Hirawaty Kamarulzaman, Hamed Norolla Bakheet Ali, Riri Amandaria, Ni Made Viantika, Aulia Nurul Hikmah

**Affiliations:** 1 Department of Magister Agribusiness, Faculty of Agriculture, Universitas Pembangunan Nasional Veteran Jawa Timur, Surabaya, Indonesia; 2 Department of Agricultural Socioeconomics, Hasanuddin University, Makassar, Indonesia; 3 Graduate Program of Resource and Environmental Economics, IPB University, Bogor, Indonesia; 4 Department of Agribusiness and Bioresource Economics, Faculty of Agriculture, Universiti Putra Malaysia, Serdang, Malaysia; 5 Agricultural Economics and Rural Development, Faculty of Agriculture, Omdurman Islamic University, Omdurman, Sudan; 6 Department of Sociology and Anthropology, University of Negeri Makassar, Makassar, Indonesia; 7 Department of Agribusiness, Faculty of Agriculture, Universitas Pembangunan Nasional Veteran Jawa Timur, Surabaya, Indonesia

**Keywords:** Geographical Indication, multi-actor collaboration, rural development, smallholder livelihoods, value chain governance

## Abstract

**Introduction:**

North Toraja's Arabica coffee sector—recognized for its distinctive quality and protected by Geographical Indication (GI) status—continues to face sustainability challenges shaped by institutional dynamics, power asymmetries, and socio-cultural contexts. Despite strong international demand, smallholder farmers contend with aging plantations, climate variability, weak bargaining power, and fragmented governance that constrain access to resources and influence over decision-making.

**Methods:**

This study adopts a systems thinking approach using interpretive structural modeling (ISM) to examine stakeholder interactions, map systemic constraints, and identify leverage points for inclusive transformation. Data were collected through in-depth interviews, expert questionnaires, and field observations involving farmers, cooperatives, traders, small and medium enterprises, and government agencies, and were supplemented with secondary sources.

**Results:**

The ISM results indicate that upstream institutional strategies—particularly minimum price guarantees and certified seed systems—exert the strongest driving power, shaping the feasibility of downstream initiatives across the value chain. Conversely, interventions such as youth engagement, post-harvest innovation, and digital marketing show high dependence on enabling governance, coordinated action, and sustained institutional support. Key barriers include intermediary dominance and the absence of sustained cross-actor collaboration.

**Discussion:**

These findings suggest that sustainable transformation requires linking top-down governance reforms with bottom-up empowerment to rebalance bargaining power and strengthen local agency. Multi-stakeholder platforms, inclusive decision-making processes, and context-sensitive institutional arrangements can improve coordination and unlock dependent initiatives. As a novel contribution, the study moves beyond descriptive stakeholder mapping by structurally ranking sustainability interventions and barriers according to their driving-dependence relationships, thereby identifying actionable institutional leverage points for GI-protected, smallholder coffee value chains. By integrating institutional, social, and economic perspectives, this study advances a sociologically grounded framework for agribusiness transformation, offering transferable insights for other smallholder-dominated commodity systems in the Global South.

## 1 Introduction

The sustainability of the agricultural commodity sectors depends on ecological, economic, and complex social factors, including institutional arrangements and power relations among actors (Barreto et al., 2023; Pratiwi et al., 2021). Toraja, Indonesia, Arabica coffee, globally recognized for its quality and protected by Geographical Indication (GI) status, serves as both a key livelihood source and a contested socio-economic space (Anhar et al., 2021; Irmayani et al., 2024; Rosiana and Feryanto, 2022). Its development is shaped by historical legacies, sociocultural norms, and institutional fragmentation, which influence how benefits, responsibilities, and decision making are distributed (Neilson and Shonk, 2014; Rosiana and Feryanto, 2022). Despite strong market demand, smallholders face low bargaining power, generational decline, and limited resources (Musa et al., 2020; Panggabean et al., 2022), whereas intermediaries' dominance, weak coordination, and limited institutional support hinder collective action (Meter et al., 2023; Sia et al., 2025; Stofya, 2023). These challenges are rooted in broader sociological dynamics, including enduring traditional authority, changing community aspirations, and differing visions of rural development (Dietz et al., 2018; Rico et al., 2024; Samper and Quiñones-Ruiz, 2017), particularly the persistence of traditional authority structures that centralize decision-making and constrain farmer agency, the shifting aspirations of younger generations who increasingly perceive farming as economically and socially unattractive, and competing visions of rural development that prioritize market integration or elite-led modernization over inclusive smallholder empowerment (Dietz et al., 2018; Rico et al., 2024; Samper and Quiñones-Ruiz, 2017).

Indonesia, one of the world's top five coffee exporters, holds strategic importance in the global coffee market through both its volume and diversity, with Arabica varieties such as Toraja coffee contributing significant value, despite comprising only 27% of the national output (Ashardiono and Trihartono, 2024; BPS, 2024). Toraja Arabica coffee, recognized for its distinct flavor profile and protected by Geographical Indication (GI), plays a critical role not only in international trade, but also in preserving local traditions and livelihoods (Anhar et al., 2021; Irmayani et al., 2024; Rugerinyange and Buyinza, 2023). However, traditional Arabica farmers face persistent barriers, including low productivity, poor infrastructure, and limited market access, which threaten their competitiveness in the specialty coffee segment (Pratiwi et al., 2021; Rico et al., 2021). Indonesia must address these constraints to sustain its global advantage through integrated strategies that combine institutional coordination, stakeholder empowerment, and investment in quality enhancement and postharvest systems (Ashardiono and Trihartono, 2024; Manalu et al., 2019). The study explores these dynamics within North Toraja's coffee agribusiness system, offering evidence-based insights into the institutional roles, systemic constraints, and strategic interventions that support inclusive and sustainable development.

Recent production data reveal marked fluctuations in North Toraja's Arabica coffee output over the past 5 years (2019–2023), peaking at 10,015.25 tons in 2022 and declining to 9,193.75 tons in 2023, highlighting the sector's vulnerability to climate variability, aging plantations, limited generational renewal, and weak value chain integration (Contreras-Medina et al., 2024, 2020; Parada-Molina et al., 2022; Peng et al., 2022). Smallholder farmers, who dominate production, face systemic barriers such as limited capital, inadequate farm management skills, and fragmented market structures dominated by intermediaries, which restrict their participation in price negotiations and sustainability initiatives and ultimately weaken their bargaining power (Musa et al., 2020; Rosiana and Feryanto, 2022; Salam et al., 2021). Despite promoting value chain development as a strategy for rural transformation, its effectiveness in Toraja remains constrained by complex local livelihoods and sociocultural dynamics (Neilson and Shonk, 2014). Consequently, researchers have stressed the need for sustainable farming practices, capacity building, and improved access to financial resources to enhance farmer welfare and Toraja's competitiveness in the specialty coffee market (Panggabean et al., 2021, 2022).

Sustainability in Arabica coffee production hinges on the interaction of ecological, economic, social, technological, and institutional dimensions shaped by diverse actors along the value chain, deeply rooted in structural constraints that transcend mere agronomic limitations (Barreto et al., 2023; Harvey et al., 2021; Pratiwi et al., 2021; Samper and Quiñones-Ruiz, 2017). However, much of the literature on Indonesian coffee agribusiness emphasizes isolated interventions, such as certification, agroforestry, and cooperative development, without addressing the systemic interdependencies influencing long-term sustainability (Meter et al., 2023; Sia et al., 2025; Stofya, 2023). Recent research argues that certification alone is insufficient and calls for more integrated approaches that consider enabling environments, market access, producer capacity, and multi-actor governance (Bashiri et al., 2021; Ibnu, 2023). The fragmented structure of Indonesia's coffee value chains and the dominance of intermediaries continue to marginalize smallholders and limit their engagement in sustainability efforts (Rosiana and Feryanto, 2022). The study adopts interpretive structural Modeling (ISM) to analyze stakeholder roles and systemic constraints in North Toraja's coffee agribusiness to identify leverage points for inclusive and coordinated sustainability pathways.

Globally, research has emphasized the importance of multi-stakeholder collaboration in achieving effective sustainability governance (Dietz et al., 2018). However, empirical studies that systematically map actor relationships and analyze the hierarchy of systemic constraints within the Indonesian coffee sector, particularly in speciality-producing regions such as North Toraja, highlight the need for collaboration. This gap is critical because the complexity of coffee agribusiness in North Toraja is shaped not only by agronomic factors but also by historical legacies, sociocultural dynamics, and fragmented institutional arrangements (Rosiana and Feryanto, 2022). Without a clear understanding of these dynamics, sustainability interventions remain fragmented and ineffective.

Researchers worldwide recognize multi-stakeholder collaboration as the key to achieving effective sustainability governance in agri-food systems (Dietz et al., 2018). However, empirical studies in Indonesia, especially in specialty coffee regions such as North Toraja, have rarely mapped the relationships between actors or assessed the hierarchical structure of systemic constraints. This is a critical gap, as the sustainability of North Toraja's Arabica coffee agribusiness is shaped not only by agronomic issues but also by historical legacies, sociocultural dynamics, and institutional fragmentation (Rosiana and Feryanto, 2022). Past efforts have often focused on single interventions, such as certification or cooperative strengthening, while overlooking how stakeholder roles intersect with broader system dynamics (Meter et al., 2023; Sia et al., 2025). Recent research emphasizes that sustainability challenges, including pest outbreaks, climate variability, and price volatility, must be addressed through coordinated, cross-sectoral strategies that center on local realities and farmer voices (Rico et al., 2024; Smith et al., 2022; Wijaya et al., 2017). The study underscores the need for a comprehensive study of stakeholder dynamics in North Toraja's Arabica coffee sector by employing interpretive structural Modeling (ISM) to map stakeholder interrelations and identify leverage points for inclusive and systemic transformation. There is a significant gap in the literature regarding comprehensive frameworks that examine the roles of key actors across sustainability dimensions and systemic barriers that hinder effective collaboration and outcomes. In particular, few studies have applied structured methods, such as interpretive structural Modeling (ISM), to map actor interdependencies and the hierarchy of constraints within a coffee agribusiness system. Addressing this gap is crucial for identifying leverage points at which targeted interventions can drive more inclusive, equitable, and sustainable development in the sector.

The study adopts a systems thinking perspective to examine sustainability challenges in North Toraja's Arabica coffee sector, viewing agribusiness systems as complex, adaptive, and interdependent rather than linear (Sterman, 2000; Merker et al., 2015; Banson et al., 2015). In smallholder-dominated coffee systems, sustainability outcomes emerge from reinforcing feedback relationships among production decisions, institutional arrangements, market power and social dynamics (Neilson and Shonk, 2014; Samper and Quiñones-Ruiz, 2017). Weak farmer bargaining power, for example, can reduce investment incentives, contributing to productivity decline and further entrenching farmers' disadvantaged market position (Rosiana and Feryanto, 2022; Vicol et al., 2018). Recognizing these reinforcing and constraining feedback mechanisms is essential for identifying leverage points that support inclusive and sustainable value chain transformation (Merker et al., 2015; Dietz et al., 2018; Rico et al., 2024).

In our study, “sustainability” refers to a development approach that simultaneously integrates economic viability, social justice, and environmental protection, in line with the “three pillars” concept (economic–social–environmental) that has become the dominant framework in sustainability literature, as well as the interpretation of the SDGs as an integrated and indivisible agenda that balances these three dimensions (Avelino et al., 2016; Purvis, 2019). We use “sustainable transformation” to emphasize that the improvements needed are not merely incremental but rather cross-actor and cross-component changes in the system/value chain (production practices, governance, market access, incentives, and power relations) that lead to a more sustainable coffee system. this interpretation is consistent with the literature on transformation/transition toward sustainability, which emphasizes fundamental changes in socio-technical systems, while placing politics, power, and governance as key elements of transformation (Avelino et al., 2016; Geels, 2019; van der Hel et al., 2016).

Operationally, “sustainable transformation” in the North Toraja Arabica coffee sector is assessed as a system-level shift reflected in two analytical lenses used in this study; such shifts typically require governance capacities that coordinate collective action and learning across scales (Dietz et al., 2003). First, we examine how sustainability is enabled through the roles and interconnections of stakeholders across ecological, economic, social, infrastructural, technological, and institutional dimensions of the coffee value chain, consistent with recent coffee supply-chain syntheses that emphasize multi-dimensional (environmental–social–economic–governance) challenges across chain stages (De Felice et al., 2025; Wright et al., 2024). Second, we identify and analyze systemic barriers that hinder the effective implementation of sustainability practices within the Arabica coffee agribusiness system; this aligns with recent evidence that sustainable agrifood value-chain transformation is often constrained by governance arrangements, value-addition structures, and smallholder-related compliance barriers (Ratna et al., 2023; Upreti et al., 2025). In this framing, transformation is not treated as a single outcome indicator but as a reconfiguration of stakeholder functions, relationships, and enabling conditions—particularly governance and incentive structures—that shape whether sustainability practices can be adopted and sustained (Avelino et al., 2016; Hale et al., 2023; van der Hel et al., 2016).

The study seeks to achieve two main objectives: first, to map the roles and interconnections of stakeholders within the coffee value chain by examining ecological, economic, social, infrastructural, technological, and institutional dimensions, and second, to identify and analyze the systemic barriers that impede the effective implementation of sustainability practices in the Arabica coffee agribusiness system.

The study significantly contributes to sustainable agriculture and rural development by advancing a system-thinking approach to coffee agribusiness transformation. This demonstrates that achieving sustainability in specialty coffee production, especially in regions such as North Toraja, requires more than fragmented interventions. This research underscores the need for coordinated, multi-actor strategies that acknowledge stakeholders' varied roles, interests, and capacities while tackling the structural constraints that limit equitable outcomes. By integrating these insights, the study offers a foundation for designing more inclusive, resilient, and context-sensitive pathways for sustainability in Arabica coffee value chains.

## 2 Methods

### 2.1 Research design

The study adopts a qualitative case study approach to examine stakeholder roles and systemic barriers in Arabica coffee agribusiness in North Toraja, Indonesia using Interpretive Structural Modeling (ISM) as the main analytical tool. The qualitative design enables an in-depth exploration of ecological, economic, social, technological, and institutional sustainability dimensions within the local value chain. ISM is particularly suitable for this study because the research objective is not only to list constraints but to reveal how multiple constraints and actors are structurally interrelated, and to identify which barriers act as key drivers vs. those that are largely dependent outcomes. In complex agri-food value chains—where ecological, economic, social, technological, and institutional factors interact—ISM provides a transparent and systematic procedure to translate expert knowledge into a hierarchical model of influence, enabling the identification of leverage points for intervention. Compared with approaches that primarily rank factors (scoring or frequency-based prioritization) or focus on pairwise causal estimation, ISM is advantageous here because it explicitly models directional relationships among elements (i → j), produces reachability and antecedent structures, and yields a driver–dependence classification that supports strategic sequencing of actions.

Primary data were collected through purposive sampling of key informants—farmers, cooperatives, traders, processors, government officials, SMEs, and others—while secondary data came from policy reports, statistics, and prior studies. Following standard ISM procedures, we elicited expert judgments to construct the Structural Self-Interaction Matrix (SSIM), converted it into reachability matrices, and derived the final hierarchical structure and driver–dependence diagram. This mixed qualitative–structuring approach strengthens methodological rigor by combining contextual depth from the case study with a replicable modeling logic, allowing the study to pinpoint leverage points and prioritize strategic interventions for more inclusive and sustainable coffee sector development.

### 2.2 Study sites

**Figure 1:**
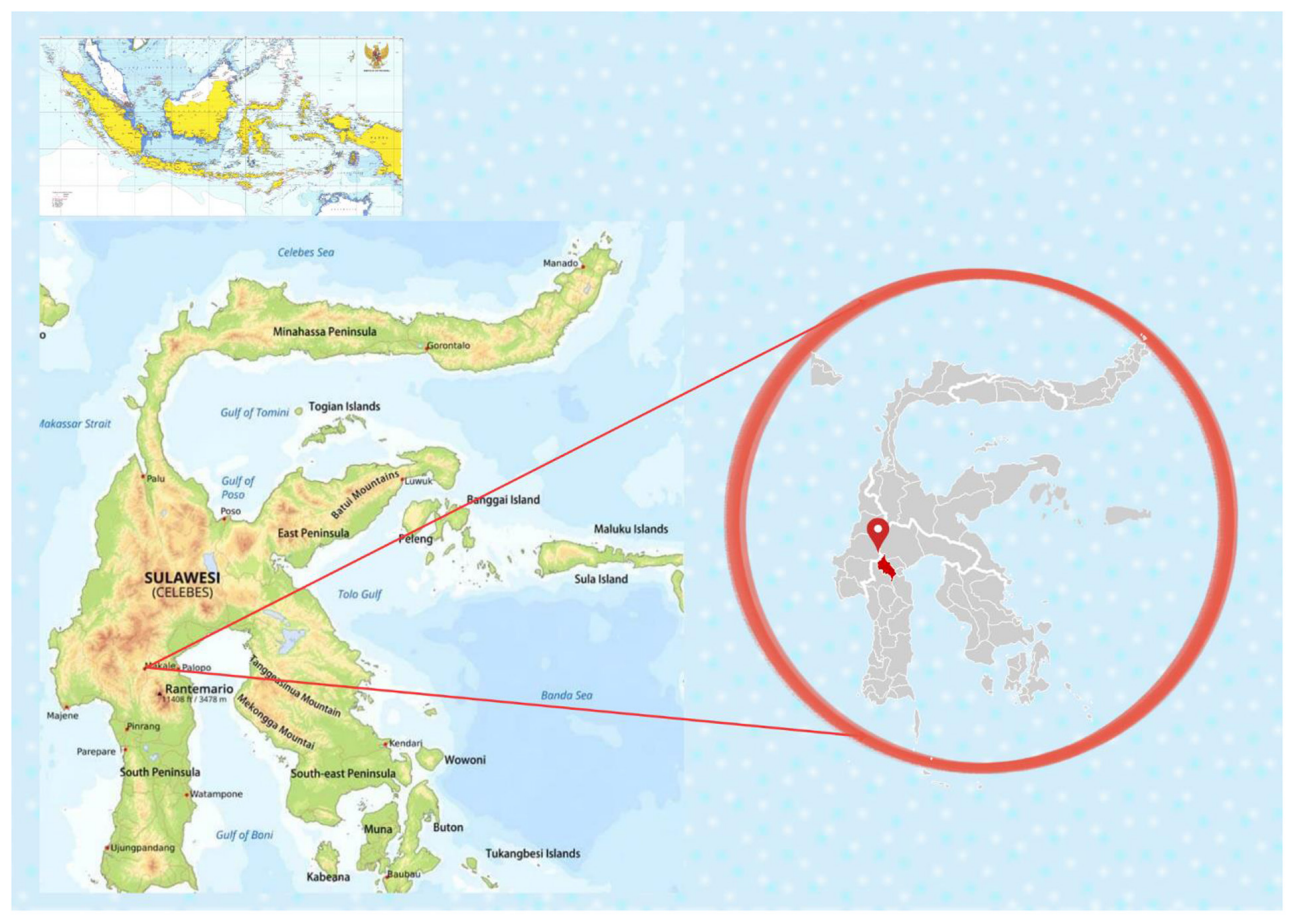
Research area in North Toraja Regency (Wikimedia, 2026a,b; Free World Map, 2026).

The research area is North Toraja Regency in South Sulawesi, Indonesia, as shown in Figure 1. North Toraja was chosen as the study site due to its strategic role as a major Arabica coffee-producing region in Indonesia, known for its high-quality speciality coffee and protected by Geographical Indication (GI) status. Its unique agro-ecological features, such as high land altitude, volcanic soil, and favorable microclimates, produce a distinctive flavor profile that is highly valued in domestic and international markets including Japan, Europe, and the United States (Irmayani et al., 2024; Rosiana and Feryanto, 2022). Despite its strong market position, the region faces ongoing challenges like aging plantations, variable productivity, limited access to premium markets, and the low bargaining power of smallholder farmers.

The value chain remains dominated by intermediaries, with minimal farmer involvement in key decisions regarding pricing, certification, and sustainability (Sia et al., 2025). These dynamics, shaped by ecological, economic, social, and institutional factors, make North Toraja an ideal case study for examining how stakeholder roles and systemic barriers affect sustainability. Its long-standing coffee heritage dating back to the colonial era and its central role in rural livelihoods further underscore its relevance for exploring inclusive and sustainable pathways in coffee agribusiness.

### 2.3 Data collection

The study employed primary and secondary data collection methods to comprehensively examine stakeholder roles and systemic barriers to Arabica coffee agribusiness in North Toraja. Primary data were gathered through semi-structured interviews, an ISM expert questionnaires, and field observations. We engaged directly with key informants representing major nodes of the local value chain, including farmers, cooperatives, traders, processors, retailers, SMEs, extension agents, and government officials from relevant agencies. This multi-source engagement strengthened contextual understanding and supported triangulation of findings.

The study selected informants through purposive sampling to ensure information-rich cases and adequate representation of upstream and downstream stakeholders. Selection was based on: (i) direct involvement in Arabica coffee production, processing, trading, service provision, or governance in North Toraja; (ii) role-based knowledge of decision-making processes and coordination mechanisms within the value chain (e.g., leadership/managerial responsibilities or institutional mandates); and (iii) demonstrated experience and familiarity with sustainability-related challenges and interventions in the sector (e.g., engagement with quality standards, GI-related practices, extension programs, cooperative management, market access, or policy implementation). Participation was voluntary and preceded by informed consent, consistent with ethical research practice.

In total, 17 key informants were recruited to balance depth and diversity while maintaining feasibility for the ISM procedure, which requires repeated expert judgments to establish pairwise directional relationships among elements. This panel size ensured coverage across key stakeholder categories and enabled convergence of judgments during the ISM steps (SSIM development, reachability matrices, and driver–dependence classification), while still allowing in-depth qualitative inquiry through interviews. The semi-structured interviews and the expert questionnaire for ISM were administered to the same panel of 17 informants. Specifically, interviews were used to elicit contextual insights regarding stakeholder roles, sustainability barriers, and collaboration opportunities, while the questionnaire was used to collect structured judgments on the direction of influence among identified elements (for constructing the SSIM and subsequent ISM matrices). This integration ensured consistency between qualitative insights and the structured modeling inputs.

The interviews used open-ended questions to explore stakeholders' roles in sustainability, barriers to progress, and strategies for improving collaboration and resilience. Field observations at farms, cooperatives, processing facilities, and coffee shops complement these insights. Secondary data—including policy reports, statistics, academic studies, and relevant documents—were also used to strengthen contextual understanding and corroborate primary findings.

The components and sub-components of the study were selected based on the goals of the investigation. They identified three key components: (1) the actors involved in the sustainability of the Arabica coffee industry, (2) barriers to this industry's sustainability, and (3) the Arabica Coffee Sustainability Strategy in North Toraja District (Details are provided in Supplementary Tables 1, 2). The study identified these aspects and sub-elements based on the study goals, analytical model, and insights gathered from discussions with specialists knowledgeable about Arabica coffee.

Informants were selected using purposive sampling based on a stakeholder map of the North Toraja Arabica Coffee value chain, with the aim of obtaining a variety of roles, levels of influence, and sectoral knowledge. Inclusion criteria included: (i) direct involvement in the value chain (production, processing, marketing, service/funding/extension support, or regulation); (ii) minimum experience (≥5–7 years) or a position that demonstrated operational understanding; (iii) decision-making/representative capacity (holding a key role in an organization/community/agency or representing a stakeholder group); and (iv) diversity of actors to cover the main categories (farmers, cooperatives, farmer groups, MSME, coffee shops, agriculture officers).

Extension agents were part of the 17 informants; because they are institutionally positioned within the district agriculture offices in North Toraja, we reported them under “agriculture officers/government officials.” They provided perspectives on advisory services, program implementation, and coordination across value-chain actors, which informed both the interviews and the ISM structuring process. On the other hand, intermediaries (local collectors/brokers) were not directly interviewed in this study. To reduce potential one-sidedness, we triangulated perspectives on intermediary roles using accounts from farmers, cooperatives, processors/SMEs, and government/extension officers, complemented by field observations and secondary sources. This design enabled us to capture how intermediary practices are perceived and experienced by multiple actors, although it does not replace intermediaries' own accounts.

### 2.4 Data analysis

#### 2.4.1 Interpretive structural modeling

The study employs interpretive structural Modeling (ISM) as the primary analytical approach to examine the interrelationships among actors, constraints, and strategies in the Arabica coffee agribusiness system in North Toraja. ISM is a well-established system-structuring method that transforms qualitative expert judgments into a hierarchical model, enabling the identification of contextual relationships and levels of influence within complex systems (Warfield, 1974; Sage, 1977; Sushil, 2012). The analysis followed established ISM procedures, beginning with the identification of key elements through a literature review, field context assessment, and expert validation, followed by defining directional relationships among elements (Attri et al., 2013; Gardas et al., 2017). Pairwise comparisons using expert questionnaires and in-depth interviews were used to construct the Structural Self-Interaction Matrix (SSIM), which was subsequently converted into the Initial Reachability Matrix (IRM) and refined into the Final Reachability Matrix (FRM) through transitivity rules to capture both direct and indirect relationships (Sushil, 2012; Govindan et al., 2010). Hierarchical level partitioning and the calculation of driver power and dependence enabled the classification of elements into autonomous, dependent, linkage, and independent categories, thereby identifying the root drivers and leverage points within the system (Mangla et al., 2016). To enhance trustworthiness, the ISM results were triangulated with field observations, secondary data, and follow-up discussions with key informants, thereby strengthening the interpretation of the hierarchical relationships that shape sustainability outcomes (Onososen and Musonda, 2022).

##### 2.4.1.1 The structural self-interaction matrix structure

Let E = {e_1_,e_2_,…,en} denote the set of elements (e.g., actors, barriers, or strategies). The Structural Self-Interaction Matrix (SSIM) is defined as S = [sij], where each entry sij ϵ {V,A,X,O} represents the contextual relationship between ei and ej, based on expert judgement:

- V: ei influences ej
- A: ej influences ei
- X: ei and ej influence each other
- O: no relation between ei and ej

##### 2.4.1.2 Transformation to initial reachability matrix

Transformation rules:


$$\begin{array}{l} IRMij\{\begin{array}{c} 1,\;if\;SSIMij=V\;or\;X\\ 0,\;if\;SSIMij=A\;or\;O \end{array}\\ IRMji\{\begin{array}{c} 1,\;if\;SSIMij=A\;or\;X\\ 0,\;if\;SSIMij=V\;or\;O \end{array} \end{array}$$

##### 2.4.1.3 Reachability and antecedent sets

The intersection of these sets determines the hierarchical level:


L (ei)=Levelk if R (ei) ∩ A (ei)=R (ei)

These elements are placed at level k, removed from further computation, and the process is repeated for the remaining elements.

#### 2.4.2 Mathematical formulation of the initial reachability matrix

##### 2.4.2.1 Transformation rules from SSIM to IRM

The SSIM was converted into the Initial Reachability Matrix (IRM), a binary matrix $R^{\left(0\right)}\;=\left[r_{ij}^{\left(0\right)}\right]\;$ε {0,1} ^*n*×*n*^ using the standard conversion rules:


$$\begin{array}{l} r_{ij}^{\left(0\right)}=\{\begin{array}{c} 1\\ 0 \end{array}\;\begin{array}{cc} If\;sij=V\;or & Sij\\ If\;sij=A\;or & Sij \end{array}\;r_{ji}^{\left(0\right)}=\{\begin{array}{c} 1\\ 0 \end{array}\;\begin{array}{cc} If\;sij=V\;or & Sij\\ If\;sij=A\;or & Sij \end{array}\;\begin{array}{cc} - & X\\ - & O \end{array} \end{array}$$

Diagonal entries were set to one for all elements:


$$\begin{array}{l} r_{ii}^{\left(0\right)}-1\;\forall i \end{array}$$

##### 2.4.2.2 Matrix form

If there are n elements, then the Initial Reachability Matrix IRM is an n × n binary matrix:


$$\begin{array}{l} IRM=\;\left[\begin{array}{ccc} IRM11 & IRM12 & \ldots \;\\ \vdots & \vdots & \ddots \\ IRMn1 & IRMn2 & \ldots \end{array}\begin{array}{c} IRM1n\\ \vdots \\ IRMnn \end{array}\right] \end{array}$$

Where each entry is filled according to the above transformation rules.

#### 2.4.3 Final reachability matrix, level partitioning, and driver–dependence

The Final Reachability Matrix (FRM), R = [rij], was obtained by incorporating transitivity (if ei → ek and ek → ej, then ei → ej). In Boolean terms, the transitive closure can be expressed as:


$$\begin{array}{l} R-\;R^{\left(0\right)}\;V\;{\left(R^{\left(0\right)}\right)}^{2}\;V\;.\;.\;.\;V\;{\left(R^{\left(0\right)}\right)}^{k} \end{array}$$

Where V denotes the Boolean OR operator, and matrix powers are computed using Boolean multiplication. The value of kkk is chosen such that no new reachability relations are added.

##### 2.4.3.1 Reachability and antecedent sets (for level partitioning)


$$\begin{array}{l} Reachability\;set\;:\;R\left(ei\right)-\;\left\{ej\;|\;r\;ij-1\right\} \end{array}$$

Elements assigned to level *k* satisfy:


$$\begin{array}{l} R\left(ei\right)\;\cap \;A\left(ei\right)=R\left(ei\right) \end{array}$$

These elements are placed at level k, removed from further iteration, and the procedure is repeated until all levels are obtained.

##### 2.4.3.2 Driver power and dependence

Driver power and dependence were computed from the FRM as row and column sums, respectively:


$$\begin{array}{l} Di=\overset{n}{\underset{j=1}{\sum }}FRMij\;Ri=\overset{n}{\underset{j=1}{\sum }}FRMji \end{array}$$

where *Di* is the driver power and *Pi* is the dependence of element *ei*. The next explanation is the Final Canonical Matrix (details are provided in Supplementary Table 3). To ensure consistency in the ISM analysis, the notation is defined in Table 1.

**Table 1:** Variables and parameters summary.

**Symbol**	**Meaning**
ei, ej	Elements (actors, barriers, or strategies)
SSIMij	Relationship between ei and ej using V, A, X, O
IRMij	Binary matrix cell (1 = relation exists, 0 = none)
R(ei)	Reachability set for element i
A(ei)	Antecedent set for element i
L(ei)	Level assignment for element i in ISM hierarchy

In improving readability, tables/figures that are detailed supporting materials have been moved to Supplementary material, while the main manuscript retains only the most essential visuals to explain stakeholder structures and key constraints.

## 3 Results

### 3.1 Actor roles, constraints, and interactions from upstream to downstream

The upstream stage of the Arabica coffee value chain in North Toraja is dominated by smallholder farmers and farmer groups, who are central to production but operate under significant constraints. These include aging plantations, limited access to certified seedlings, climate-induced production variability, and a declining interest among younger generations in engaging in coffee farming. Extension services and local government agencies play a supporting role at this stage; however, their influence is often constrained by limited resources and fragmented coordination. As a result, farmers tend to occupy a highly dependent position within the system, despite their critical role in sustaining coffee production and maintaining the Geographical Indication (GI) reputation of Toraja coffee.

Moving downstream to the midstream stage, cooperatives, local traders, and small and medium enterprises emerge as key intermediaries linking farm-level production to markets. This stage encompasses post-harvest handling, processing, storage, and quality control, where coordination failures and uneven technology adoption significantly affect the retention of value. Cooperatives have the potential to strengthen farmers' bargaining power and improve quality consistency, but their effectiveness varies depending on managerial capacity, member participation, and alignment with buyer requirements. Local traders often fill coordination gaps; however, their dominance can reinforce power asymmetries, limit transparency, and weaken farmers' influence over price formation and quality standards.

In the downstream stage, companies, exporters, and specialty market actors exert substantial influence over governance, certification, and value capture. Decisions made at this stage, such as pricing mechanisms, quality specifications, and market access requirements, feed back into upstream and midstream behaviors, shaping production incentives and investment decisions. The interaction across stages reveals reinforcing and constraining relationships: weak bargaining power upstream reduces incentives for quality upgrading, while limited post-harvest capacity midstream constrains access to high-value downstream markets. These interdependencies highlight that sustainability challenges cannot be addressed in isolation at any single stage, underscoring the importance of coordinated interventions and inclusive governance mechanisms that align actor roles and incentives across the entire value chain.

These upstream–downstream insights were triangulated with field observations across production and post-harvest sites and with secondary sources (policy documents, statistics, and prior studies), which corroborated the reported production constraints, coordination gaps, and uneven technology adoption described by informants.

### 3.2 Roles and hierarchical structure of key actors in the coffee agribusiness

Companies and cooperatives play a central role in shaping the sustainability of the Arabica coffee value chain in North Toraja, holding substantial influence across the system. The Agriculture Office, extension workers, traders, and MSMEs act as intermediaries facilitating stakeholder coordination. Despite being institutionally dependent, farmers and farmer groups are key drivers of sustainability, highlighting the need for both top-down support and bottom-up engagement. In contrast, technical actors, such as the chemical input and machinery industries, occupy a more peripheral role in the system.

Table 2 indicates that companies (A1) and cooperatives (A2) are the most influential actors in North Toraja's Arabica coffee value chain, with high dependence and strong driving power, positioning them as the key drivers of sustainability. The Agriculture Office (A8) coordinates, with coffee shops, MSMEs, traders, and extension workers serving as intermediaries. Notably, despite their high dependence, farmers (A11) and farmer groups (A10) possessed the strongest driving power, emphasizing their vital role in enabling change. In contrast, the chemical input (A9) and machinery (A7) industries have limited influence, reflecting their peripheral positions in the system.

**Table 2:** Actors final canonical matrix.

**Code**	**A1**	**A2**	**A3**	**A4**	**A5**	**A6**	**A7**	**A8**	**A9**	**A10**	**A11**	**D-P**	**Rank**	**D**	**Hierarchy**
A1	1	1	1	1	1	1	1	1	1	1	1	11	1	8	2
A2	1	1	1	1	1	1	1	1	1	1	1	11	1	5	5
A3	0	0	1	1	1	1	0	1	1	1	1	8	3	7	3
A4	0	0	1	1	0	1	0	1	1	1	1	7	4	8	2
A5	1	1	1	0	1	0	0	0	0	1	1	6	5	8	2
A6	0	0	1	1	0	1	0	1	1	1	1	7	4	8	2
A7	1	1	0	0	0	0	1	0	0	1	1	5	6	6	4
A8	1	0	1	1	1	1	1	1	0	1	1	9	2	7	3
A9	1	0	0	0	1	0	0	0	1	1	1	5	6	6	4
A10	1	1	0	1	1	1	1	0	0	1	1	8	3	11	1
A11	1	0	0	1	1	1	1	1	0	1	1	8	3	11	1

D-P, Driver-Power; D, Dependence.

### 3.3 Actor influence and dependency: directional analysis (DP-D)

The actor's direction chart (DP-D) illustrates the stakeholder roles in North Toraja's coffee agribusiness system, based on their driving and dependence power. Companies and cooperatives have emerged as independent actors with high influence and low dependence, underscoring their central role in advancing sustainability. Farmers and farmer groups occupy the linkage quadrant, reflecting their dual roles in influencing and being influenced, positioning them as key change agents. In contrast, coffee shops, local traders, and MSMEs are dependent actors with limited influence, whereas the chemical input and machinery industries appear autonomous with minimal connections. These insights highlight the need for stronger collaboration and empowerment of farmers to support sustainable development.

**Figure 2:**
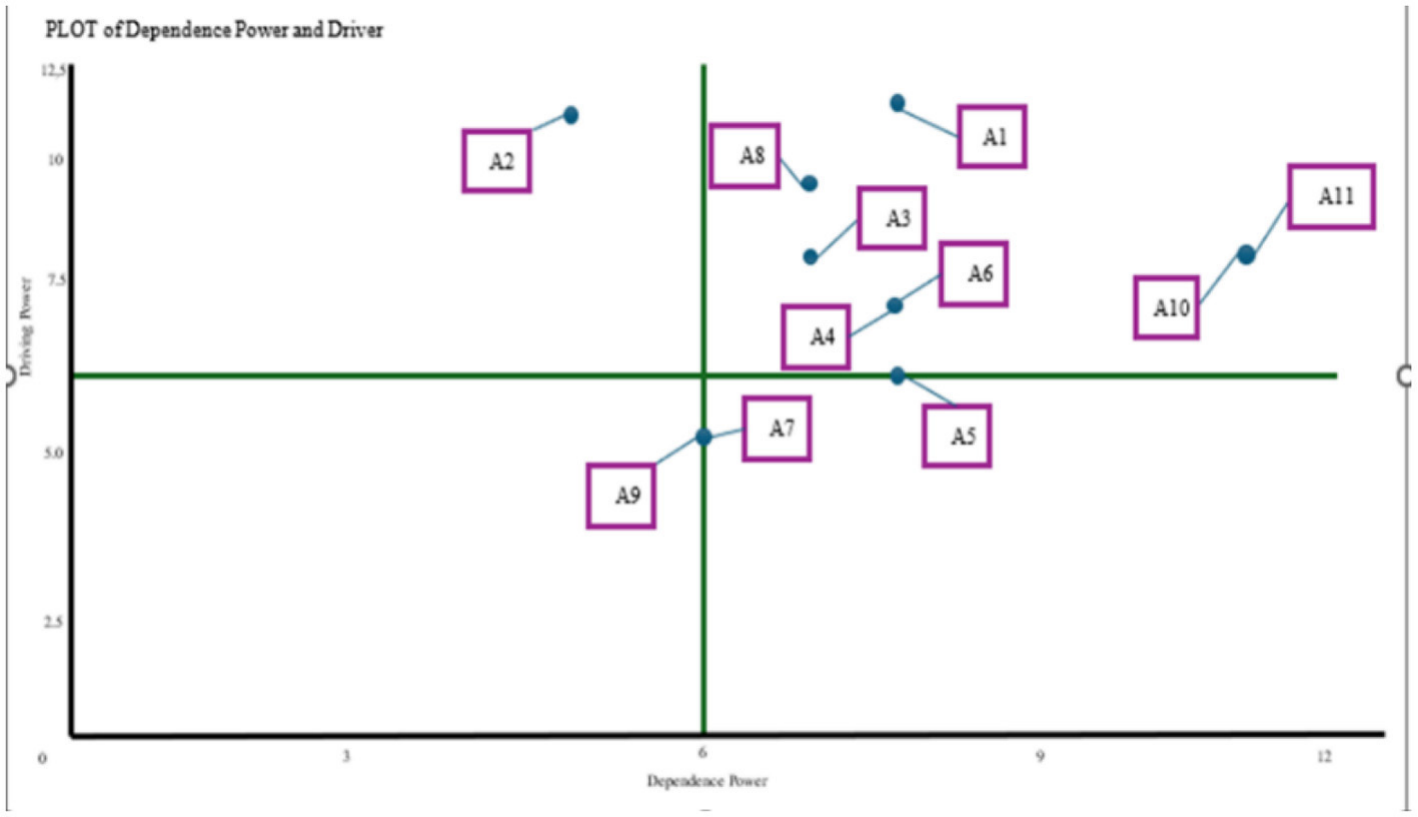
Actors' Direction Chart (DP-D) for the sustainability of Nort Toraja District's coffee agribusiness. Description: A1, Company; A2, Cooperative; A3, Coffeeshop; A4, MSME; A5, Local Trader; A6, Agricultural Extension Worker; A7, Agricultural Machinery Industry; A8, Agriculture Office; A9, Chemical Input Industry; A10, Farmer Group; A11, Farmers.

Figure 2 shows the actor's direction chart (DP-D), which maps stakeholders in the coffee agribusiness system according to their driving and dependence power. Companies (A1) and cooperatives (A2) strongly influence and minimally rely on others, positioning them as independent actors. Farmers (A11) and farmer groups (A10) fall within the linkage quadrant, reflecting their dual roles as influencers and those influenced, making them key change agents. In contrast, coffee shops (A3), local traders (A5), and MSMEs (A4) are more dependent and have limited influence, whereas the chemical input (A9) and machinery (A7) industries appear relatively disconnected. This chart underscores the need to strengthen stakeholder collaboration because key grassroots actors—farmers (A11) and farmer groups (A10)—are positioned in the linkage quadrant (high driving power and high dependence power). In ISM terms, linkage actors can catalyse change but are also sensitive to constraints and actions from other stakeholders; therefore, interventions targeting A10–A11 require coordinated support across the value chain (e.g., from cooperatives/companies, extension services, and government). Empowering farmers—through strengthened collective organization, access to information and resources, and a greater role in decision-making—helps translate their driving potential into sustained system-wide improvements.

### 3.4 Structural positioning of actors in the sustainability framework

The hierarchical structure of actors in North Toraja's Arabica coffee agribusiness reveals that companies and cooperatives have the most significant influence. Mid-level actors, including the Agriculture Office, coffee shops, MSMEs, local traders, and extension workers, serve as intermediaries connecting top-level stakeholders with grassroots actors. Farmers and farmer groups at the base, despite being highly influenced by others, are essential to implementing sustainability efforts. This structure highlights the importance of combining strong top-down support and active bottom-up engagement to achieve meaningful and lasting sustainability.

Supplementary Figure 1 illustrates the hierarchical structure of actors involved in the sustainability of Arabica coffee agribusiness in North Toraja District. At the top are companies (A1) and cooperatives (A2), reflecting their significant influence on the system. The middle level includes the Agriculture Office (A8), coffee shops (A3), MSMEs (A4), local traders (A5), and extension workers (A6), who act as intermediaries, linking influential actors with those at the grassroots level. At the bottom are farmers (A11) and farmer groups (A10), which, despite being highly influenced by others, play a pivotal role in implementing sustainability efforts. This structure underscores the urgent need for top-down support and participation to achieve meaningful sustainability outcomes, highlighting the importance of each actor's role in the process.

### 3.5 Systemic constraints hindering sustainability

The main constraints affecting the sustainability of Arabica coffee agribusiness in northern Toraja are the dominance of elderly farmers, aging coffee plants, and climate-related production variability. These have the highest driving power and serve as root causes that influence numerous other issues. By contrast, limited access to financing and the limited APBD budget are highly dependent constraints, reflecting their emergence from deeper systemic challenges. Traditional post-harvest technology shows moderate dependence but low influence, meaning other constraints shape it yet play a limited role in driving broader system changes.

Table 3 indicates that the most influential constraints on the sustainability of Arabica coffee agribusiness in North Toraja are the domination of elderly farmers (C1), aging coffee plants (C3), and climate-related production variability (C4), each with the highest driver power (D-P = 11). These represent systemic, root-level problems that impact numerous other constraints. In contrast, limited access to financing (C7) and the limited APBD budget for coffee assistance (C10) show high dependence, suggesting they are outcomes rather than causes. Traditional post-harvest technology (C6) is moderately dependent but has low driving power, meaning it is shaped by other issues but has limited influence on the broader system.

**Table 3:** Constraints final canonical matrix.

**Code**	**C1**	**C2**	**C3**	**C4**	**C5**	**C6**	**C7**	**C8**	**C9**	**C10**	**C11**	**C12**	**D-P**	**Rank**	**D**	**Hierarchy**
C1	1	0	1	1	1	1	1	1	1	1	1	1	11	1	8	5
C2	0	1	0	0	1	1	0	1	1	1	1	1	8	3	2	8
C3	1	0	1	1	1	1	1	1	1	1	1	1	11	1	7	6
C4	1	1	1	1	1	1	0	1	1	1	1	1	11	1	8	5
C5	1	0	1	1	1	1	0	0	1	1	1	1	9	2	10	3
C6	0	0	0	1	1	1	0	0	1	1	1	0	6	5	9	4
C7	1	0	0	0	0	0	1	1	1	1	1	1	6	5	3	7
C8	1	0	1	1	1	0	0	1	1	1	1	1	9	2	8	5
C9	1	1	1	1	1	1	0	0	1	1	1	1	8	3	9	4
C10	0	0	1	0	0	1	0	0	0	1	1	1	5	6	11	2
C11	1	0	0	1	1	0	0	1	0	1	1	1	7	4	12	1
C12	0	0	1	0	1	1	0	1	0	1	1	1	7	4	11	2

D-P, Driver-Power; D, Dependence.

### 3.6 Directional mapping of constraints and their interlinkages

The ISM-based Direction Chart (DP-D) maps the key barriers to sustainability in North Toraja's coffee agribusiness, based on their driving and dependence power. Root constraints, such as the dominance of elderly farmers, aging coffee plants, and climate-related production variability, were placed in the independent quadrant, showing their strong influence on other issues. By contrast, financial limitations, weak bargaining power, and limited digital marketing capacity fall into the dependent quadrant, reflecting their status as outcomes of deeper challenges. Traditional post-harvest technology and a low interest in farmer regeneration are positioned in the linkage quadrant, influencing and being influenced by other constraints. This analysis highlights the importance of addressing the root causes to support long-term sustainability.

**Figure 3:**
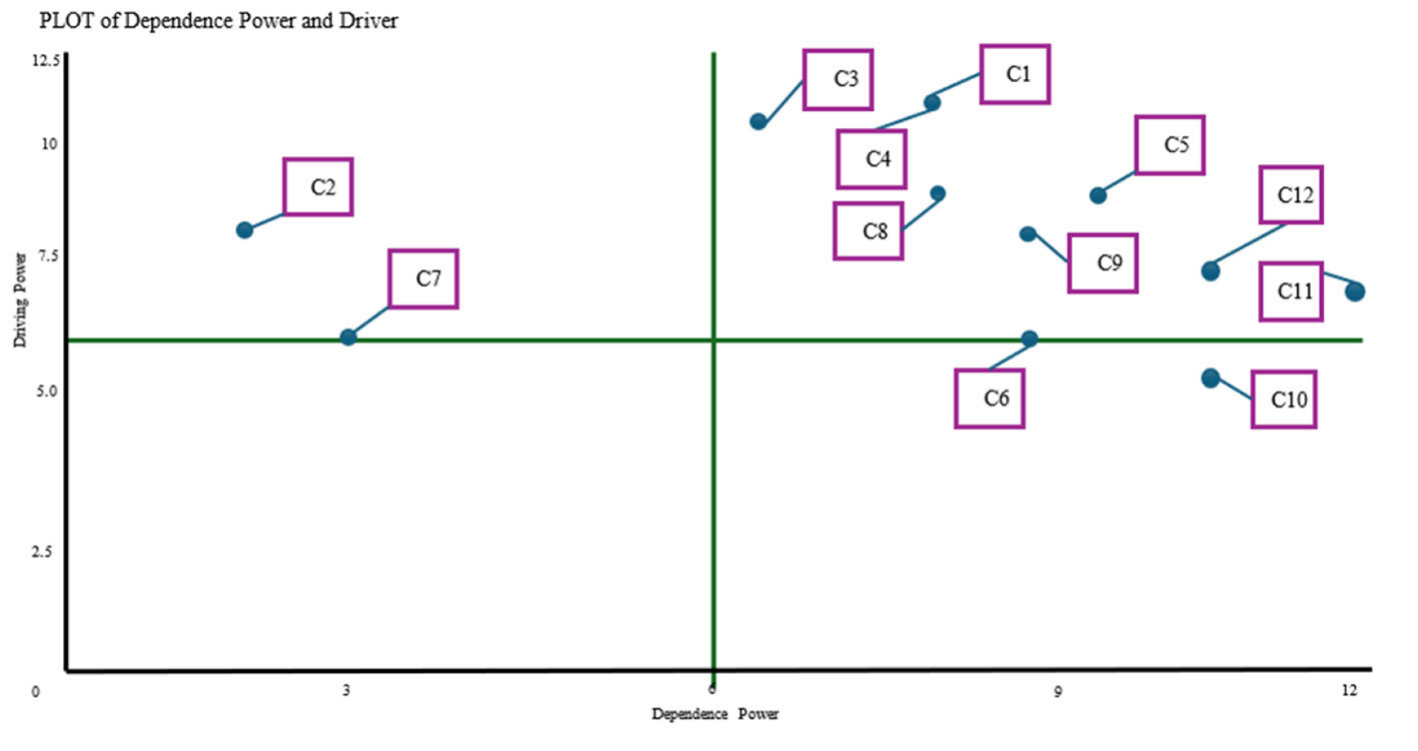
Graph of ISM for the Direction Chart (DP-D) of constraints in the sustainability of coffee agribusiness in North Toraja District. Description: C1, Domination of elderly coffee farmers; C2, Farmer group coaching is not optimal; C3, Coffee plants that have passed the optimal productive period >20 years; C4, Coffee production variability due to climate change impacts; C5, Low interest in coffee farmer regeneration; C6, Traditional post-harvest technology; C7, Limited access to financing; C8, Weak bargaining position of farmers in coffee price determination; C9, Seedling growth failure; C10, Limited APBD budget for coffee assistance; C11, Digital marketing of farmers is not yet developed; C12, Inter-regional coffee bean blending practices.

Figure 3 presents the ISM-based Direction Chart (DP-D) for constraints, illustrating their driving and dependence power in the context of coffee agribusiness sustainability in North Toraja. Core root constraints, such as the domination of elderly farmers (C1), aging coffee plants (C3), and climate-related production variability (C4), are positioned in the independent quadrant, indicating high driving power and minimal dependence, making them the primary sources of other issues. Constraints, such as financial limitations (C7), weak bargaining power (C8), and underdeveloped digital marketing (C11), appear in the dependent quadrant, reflecting their emergence from deeper systemic problems. In the linkage quadrant, traditional post-harvest technology (C6) and low interest in farmer regeneration (C5) are influenced by other constraints. This analysis highlights the importance of addressing the root causes to achieve meaningful and lasting sustainability.3.6. Hierarchical Structure of Constraints Impacting Coffee Agribusiness.

The hierarchical structure of constraints affecting Arabica coffee sustainability highlights that core issues, such as the domination of elderly farmers, aging coffee plants, and climate-related production variability, are root constraints with strong driving power. These foundational problems lead to mid-level challenges, including low interest in farmer regeneration, traditional postharvest practices, and seedling failure, contributing to dependent issues. At the bottom are constraints, such as limited financing, weak bargaining power, limited government support, and underdeveloped digital marketing, all of which stem from upstream challenges. This structure underscores the need to first address root constraints to enable broader and more effective sustainability efforts.

Supplementary Figure 2 illustrates the hierarchical structure of the constraints affecting the sustainability of Arabica coffee production in North Toraja. At the top are root constraints such as the dominance of elderly farmers (C1), aging coffee plants (C3), and climate-related production variability (C4), which have high driving power and significantly influence other issues. Mid-level constraints, including low interest in farmer regeneration (C5), traditional post-harvest methods (C6), and seedling failure (C9), stem from these root problems, while also contributing to further challenges. At the base are dependent constraints, such as limited financing (C7), weak bargaining power (C8), limited government budget (C10), and underdeveloped digital marketing (C11), which are consequences of systemic issues. This hierarchy underscores the importance of first addressing root constraints to enhance overall sustainability.

### 3.7 Strategic interventions for sustainable coffee development

The final canonical matrix of strategies shows a clear hierarchy, with upstream policy interventions, such as guaranteed price policy and seed certification, being top priorities owing to their strong influence on the overall sustainability framework. In contrast, downstream strategies, such as post-harvest technology, youth engagement, and infrastructure development, are highly dependent on these core interventions. This highlights the importance of a phased and integrated approach, in which foundational strategies serve as the backbone for broader transformation across technical, institutional, and socioeconomic areas.

Table 4 shows that the most influential strategies for supporting the sustainability of Arabica coffee agribusiness in North Toraja are the basic coffee price policy (S1) and seed certification (S2), both with the highest driving power (D-P = 14) and low dependence. These two serve as key enablers that impact all the other strategies in the system. Other important strategies—such as technical assistance (S5), digital marketing (S6), and export partnerships (S7)—also have strong driver power (D-P = 9–11), supporting dependent strategies like youth farmer education (S11), adoption of sorting machines (S12), and coffee rejuvenation (S14), which all show high dependence (D = 12–14) and rely heavily on these core interventions.

**Table 4:** Final canonical matrix of strategy.

**Strategy (Row)**	**S1**	**S2**	**S3**	**S4**	**S5**	**S6**	**S7**	**S8**	**S9**	**S10**	**S11**	**S12**	**S13**	**S14**	**D-P**	**Rank**	**D**	**Hierarchy**
S1	1	1	1	1	1	1	1	1	1	1	1	1	1	1	14	1	7	6
S2	1	1	1	1	1	1	1	1	1	1	1	1	1	1	14	1	10	4
S3	0	0	1	0	0	0	1	1	1	0	1	1	1	1	8	5	8	5
S4	0	1	0	1	0	1	1	0	0	0	0	1	1	1	7	6	6	7
S5	0	1	0	0	1	1	1	1	1	1	1	1	1	1	11	2	7	6
S6	1	0	1	0	0	1	1	1	0	0	1	1	1	1	9	4	7	6
S7	1	1	1	0	0	0	1	1	1	0	1	1	1	1	10	3	12	2
S8	1	1	1	0	0	0	1	1	1	0	1	1	1	1	10	3	11	3
S9	0	1	1	0	1	0	0	1	1	1	1	1	1	1	9	4	10	4
S10	0	1	0	0	1	0	0	0	0	1	1	1	1	1	7	6	7	6
S11	1	1	0	1	0	0	1	0	1	1	1	1	1	1	10	3	12	2
S12	0	0	0	1	1	1	1	1	1	0	1	1	1	1	10	3	14	1
S13	0	0	1	1	0	0	1	1	1	1	1	1	1	1	10	3	14	1
S14	1	1	0	0	1	1	1	1	0	1	0	1	1	1	10	3	14	1

D-P, Driver-Power; D, Dependence.

### 3.8 Directional power and dependence of strategic programs

Foundational strategies, such as the basic coffee price policy (S1) and seed certification (S2), are located in the independent quadrant, reflecting their strong influence and minimal reliance on other strategies. This positioning highlights their vital role as entry points that reassure stakeholders about the sector's stability. In the linkage quadrant are strategies like technical assistance (S5), digital marketing (S6), and export partnerships (S7), which both influence and are influenced by other programs. Youth farmer education (S11), coffee rejuvenation (S14), and postharvest technology (S12) fall within the dependent quadrant, as their success hinges on the effectiveness of prior interventions. These relationships emphasize the importance of initiating high-impact strategies to enable broader and more sustainable transformation throughout the coffee value chain.

**Figure 4:**
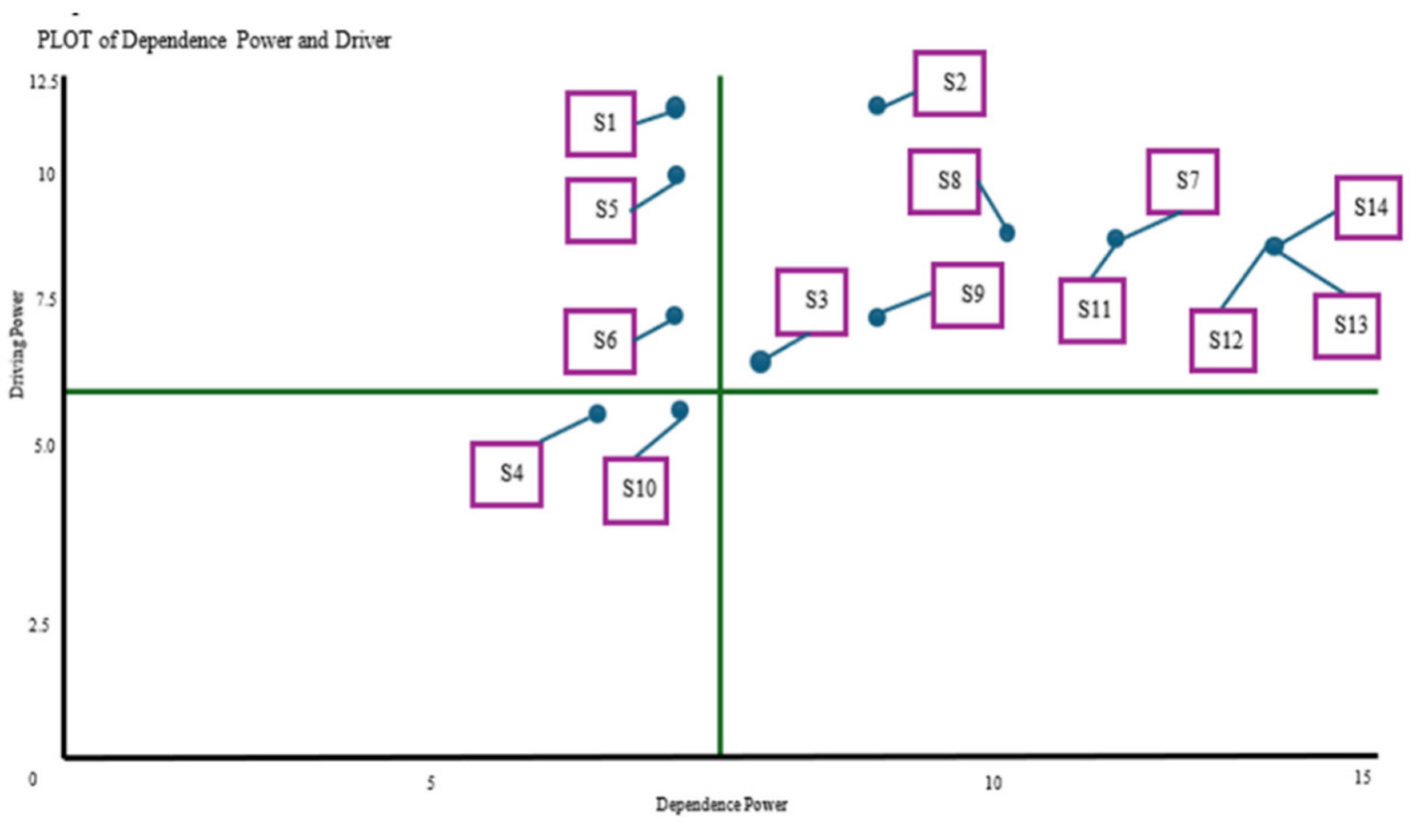
Direction graph (DP-D) of strategic program for Arabica coffee agribusiness sustainability in North Toraja District. Description: S1, Basic coffee price policy secured; S2, Arabica coffee plant seed certification; S3, Construction of collective coffee storage warehouse; S4, Drafting of PERDA Regional Regulation on Sustainable Coffee; S5, Sustainable technical assistance program for 5 years cultivation, post-harvest, and marketing; S6, Coffee Digital Marketing Innovation; S7, Sustainable export partnerships; S8, Establishment of a multistakeholder platform; S9, Integrated harvest transportation services; S10, Development of Agro-tourism-based Coffee Village; S11, Millennial coffee farmer education program Young Coffeepreneur; S12, Adoption of an automatic coffee sorting machine; S13, Provision of standardized coffee drying houses; S14, Coffee rejuvenation program based on superior local seeds.

Figure 4 shows the Direction Graph (DP-D) of strategic programs for Arabica coffee sustainability in North Toraja, mapping each strategy by its driving and dependence power. Basic coffee price policy (S1) and seed certification (S2) appear in the independent quadrant, indicating strong influence and minimal dependence, thus positioning them as priority actions. Technical assistance (S5), digital marketing (S6), and export partnerships (S7) fall into the linkage quadrant, reflecting the dual role of influencing and being influenced by other strategies. In contrast, youth farmer education (S11), coffee rejuvenation (S14), and postharvest technology (S12) are placed in the dependent quadrant, relying on the success of preceding strategies. This mapping highlights the critical roles of S1 and S2 as foundational steps for sustainable development in the coffee sector.

### 3.9 Hierarchy of strategies for Arabica coffee sustainability

The hierarchical structure of strategic programs for Arabica coffee sustainability in North Toraja is a blueprint for their effective implementation. Core strategies, such as the basic coffee price policy (S1) and seed certification (S2), sit at the top, possess the highest driving power, and guide the overall direction of the sustainability agenda. Mid-level strategies such as technical assistance (S5), digital marketing (S6), and export partnerships (S7) function as vital links between these foundational actions and more dependent initiatives. Strategies such as youth farmer education (S11), sorting machine adoption (S12), and coffee rejuvenation (S14) depend on the successful rollout of earlier programs. This structure highlights the prioritization of high-impact interventions to build a strong and sustainable foundation.

Supplementary Figure 3 depicts the hierarchical structure and implementation sequence of the strategic programs for Arabica coffee sustainability in North Toraja. At the top are the core strategies with the highest driving power, such as the basic coffee price policy (S1) and seed certification (S2), which influence all other initiatives. Midlevel strategies, including technical assistance (S5), digital marketing (S6), and export partnerships (S7), serve as bridges between foundational and dependent actions. Positioned at the bottom are strategies like youth farmer education (S11), sorting machine adoption (S12), and coffee rejuvenation (S14), which rely on the success of the preceding steps. This hierarchy underscores the importance of prioritizing high-impact strategies to establish a strong base for sustainable development.

**Figure 5:**
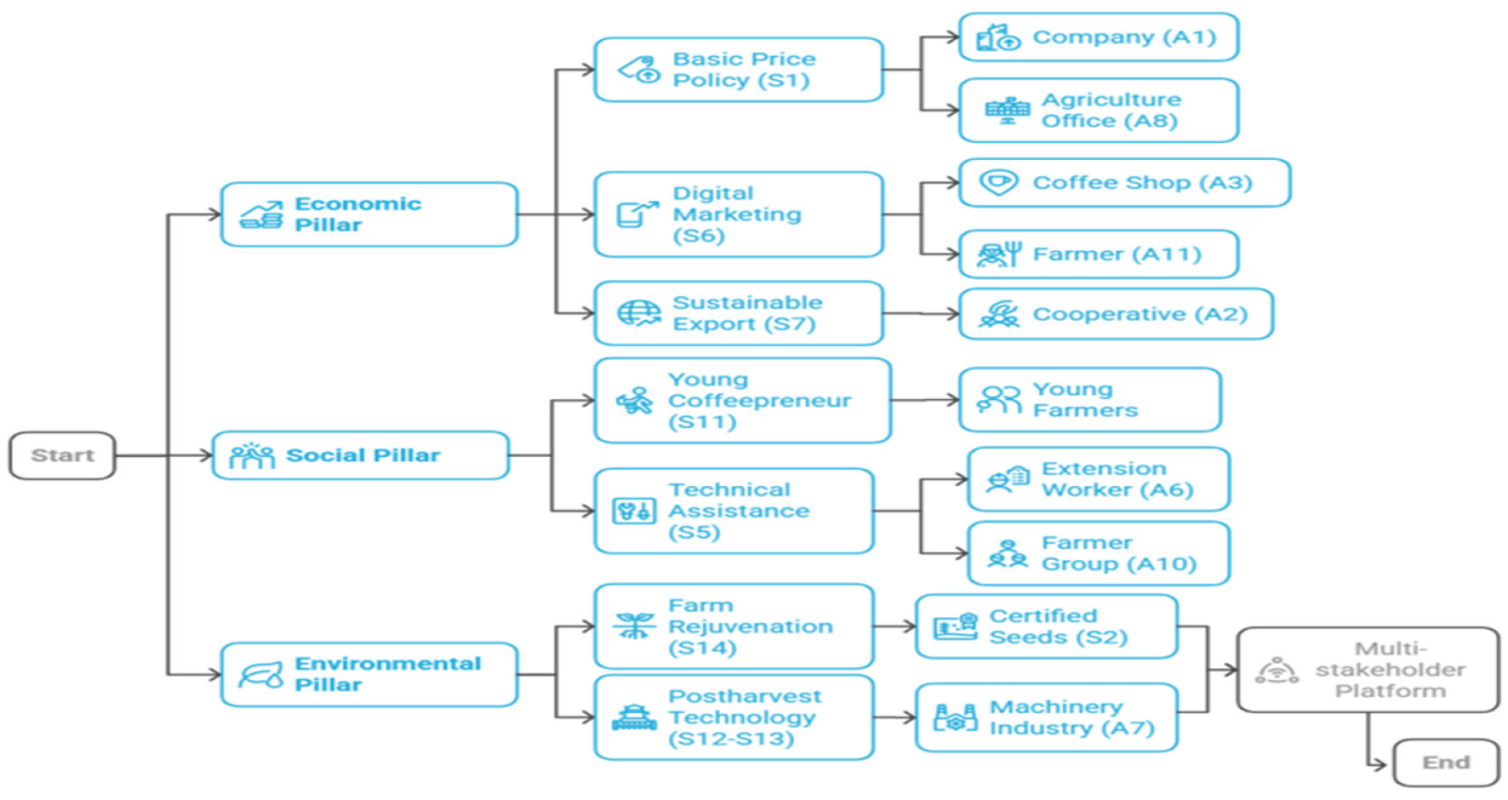
Scheme of Arabica coffee sustainability modeling. A, Actors; C, Constraints; S, Strategy.

Figure 5 presents an integrated sustainability model for Arabica coffee in North Toraja that connects key actors, constraints, and strategic interventions through a systems thinking perspective. The model was built around three components: input, process, and output. In the input phase, high-influence actors, such as companies (A1), cooperatives (A2), and farmer groups (A10), along with high-dependence actors, such as farmers (A11) and the Agriculture Office (A8), were identified (Table 3). Five major constraints (C1, C3, C4, C5, and C8) and seven impactful strategies (S1, S2, S5, S7, S8, S11, and S14) were mapped based on the stakeholder input and ISM analysis. The process stage, with multi-stakeholder collaboration (S8) as its foundation, includes three sequential phases in a structured approach: resolving key constraints (S14, S11), increasing economic value (S1, S6, S7), and enhancing institutional capacity (S4, S8). This structured approach leads to three interlinked sustainability outcomes: economic (e.g., price stability and export growth), social (e.g., youth involvement and improved skills), and environmental (e.g., farm productivity and climate resilience) presents an integrated sustainability model for Arabica coffee in North Toraja that connects key actors, constraints, and strategic interventions through a systems thinking lens. The model is built around three components—input, process, and output. In the input phase, high-influence actors such as companies (A1), cooperatives (A2), and farmer groups (A10), along with high-dependence actors like farmers (A11) and the Agriculture Office (A8), are identified (Table 3). Five major constraints (C1, C3, C4, C5, and C8) and seven impactful strategies (S1, S2, S5, S7, S8, S11, and S14) were mapped based on stakeholder input and ISM analysis. The process stage, with multistakeholder collaboration (S8) as its foundation, guides three sequential phases in a structured approach: resolving key constraints (S14, S11), increasing economic value (S1, S6, S7), and enhancing institutional capacity (S4, S8). This structured approach leads to three interlinked sustainability outcomes—economic (e.g., price stability, export growth), social (e.g., youth involvement, improved skills), and environmental (e.g., farm productivity, climate resilience).

## 4 Discussion

This study contributes to sustainability and the sustainable transformation of North Toraja's Arabica coffee sector by providing a systems-based explanation of where change should start, who must be engaged, and which interventions have the greatest leverage to shift the value chain toward more resilient and inclusive outcomes. Specifically, the ISM results identify (i) the governance configuration of actors (companies/cooperatives as high-influence actors and farmers/farmer groups as high-leverage but highly interdependent linkage actors), (ii) the root constraints that generate downstream symptoms (e.g., aging farmers/trees and climate-related variability), and (iii) a hierarchy of strategies that clarifies sequencing (foundational policies such as minimum price assurance and seed certification enabling dependent upgrading programs such as post-harvest improvement, youth engagement, and digital marketing).

These findings translate into sustainability gains across dimensions: economic sustainability through improved price stability, bargaining position, and market access; social sustainability through farmer empowerment, strengthened collective organization, and regeneration pathways for youth participation; environmental sustainability through productivity recovery, farm rejuvenation, and climate resilience; and institutional sustainability through clearer coordination, multi-stakeholder platforms, and locally embedded regulatory instruments (e.g., PERDA) that stabilize commitments over time. In this sense, “sustainable transformation” is operationalized as a coordinated, phased transition that addresses root causes first, aligns actor incentives across the chain, and converts farmers' driving potential into sustained system-wide improvements.

### 4.1 Reframing stakeholder roles in a fragmented coffee value chain

In North Toraja's Arabica coffee agribusiness, smallholder farmers are essential yet vulnerable actors facing systemic barriers, such as aging plantations, climate variability, limited credit access, and weak bargaining power (Barreto et al., 2023; Irmayani et al., 2024). ISM analysis positions farmers (A11) and farmer groups (A10) in the linkage quadrant, indicating strong driving potential alongside high dependence on other actors and system conditions within the influence–dependency matrix, reflecting broader issues in complex value chains where smallholders often lack influence and are especially exposed to climate and socioeconomic risks (Morales et al., 2022; Rico et al., 2024; Sianipar, 2012). Empowering farmers through institutional support, inclusive governance, and a territorial system perspective can enhance their adaptive capacity and support long-term value chain sustainability (Morales et al., 2022; Panggabean et al., 2022).

Farmer empowerment strategies, such as group strengthening, agribusiness training, and access to resources, capital, and infrastructure, have proven to be effective in improving productivity and income (Dzakiroh et al., 2021; Warnaen et al., 2020). However, local social and cultural contexts strongly influence the adoption of sustainable practices, such as agroforestry, so practitioners must tailor these approaches accordingly (Wienhold and Goulao, 2023). Evidence suggests that training and extension services may yield greater impact than institutional reforms alone, highlighting the need for context-sensitive approaches (Hanggana, 2024).

Enhancing farmers' voices and agencies in governance is critical. Currently, traders and companies dominate price setting and certification, limiting farmers' negotiating power (Rosiana and Feryanto, 2022; Meter et al., 2023). Establishing inclusive multi-stakeholder platforms (A8) can support dialogue, transparency, and shared decision making among farmers, cooperatives, buyers, and government actors (Dietz et al., 2018). These platforms reduce information asymmetry and build a collective capacity for advocacy and market participation.

Shifting farmers from dependency on influence also requires improved access to markets and digital tools. Digital marketing systems (A6), traceability, and quality-based incentives can link farmers directly to specialty markets, while collective storage warehouses (A3) and standardized drying facilities (A13) improve postharvest quality and bargaining power. Digital innovations enhance income by improving access to market information (Panggabean et al., 2022), while direct trade relationships and complementary strategies such as organic farming, skill development, and financial access strengthen economic viability (Borrella et al., 2015; Musa et al., 2020; Pingali et al., 2019).

Ultimately, empowering farmers is a matter of equity and a strategic necessity. As the main holders of labor and local knowledge, their active involvement is essential for scaling sustainable practices and ensuring long-term resilience. The study advocates for farmer-centric development strategies focused on capacity-building, institutional inclusion, and equitable market access, repositioning farmers as active co-creators of value in transforming North Toraja's coffee sector.

### 4.2 Addressing structural constraints through root-cause interventions

The sustainability challenges in North Toraja's Arabica coffee production stem from deeply embedded structural constraints beyond agronomic issues (Pradita and Sukardi, 2024; Harvey et al., 2023). These include aging plantations, climate-induced yield variability, market asymmetries, and weak farmer bargaining power within fragmented value chains dominated by intermediaries (Contreras-Medina et al., 2024; Irmayani et al., 2024). Underdeveloped institutional support, such as inadequate extension services, limited financing, and ineffective regeneration programs, exacerbates these problems (Stofya, 2023; Teferi, 2019). Rather than treating symptoms in isolation, sustainable solutions must address root causes such as generational disengagement, technological stagnation, and institutional fragmentation.

Thus, a multitiered intervention strategy is required. First, rejuvenating farmer demographics is critical. Programs like “Young Coffeepreneur” can help attract youth into coffee farming and foster intergenerational knowledge transfer (Sia et al., 2025). Second, all stakeholders can actively participate in improving productivity through certified seed systems, the rejuvenation of aging plantations, and post-harvest modernization. Third, institutional reforms should establish multi-stakeholder platforms that promote transparent price setting, cooperative strengthening, and inclusive decision making (Dietz et al., 2018; Meter et al., 2023), making each stakeholder feel valued and integral to the process.

Stakeholder mapping shows that while companies (A1) and cooperatives (A2) have substantial influence, farmers (A11) and farmer groups (A10) remain highly dependent, yet central to sustainability outcomes (Haslinda et al., 2024). This imbalance calls for inclusive governance reforms to empower grassroot actors. Policy tools such as guaranteed minimum prices (A1), coffee seed certification (A2), and digital marketing platforms (A6) can help transform farmers from passive price-takers into active market participants (Harvey et al., 2021; Rosiana and Feryanto, 2022).

Addressing broader sectoral constraints requires addressing weak institutional roles, fragmented policies, and limited coordination. Government agencies must provide technical support and ensure local regulations, such as PERDA (Regional Regulation) on Sustainable Coffee, and institutionalize sustainability commitments (Irmayani et al., 2024). Complementing these with investments in shared storage warehouses (A3) and standardized drying houses (A13) can reduce post-harvest losses and transaction costs. Stronger regulatory frameworks and institutional coordination are essential for aligning local initiatives with global sustainability standards (Ardana, 2019; Arifin, 2010; Ibnu, 2023; Ibnu et al., 2016; Jaya et al., 2024).

Improving sustainability in Indonesia's coffee sector, especially under Geographical Indications (GI), demands multidimensional reforms across ecology, economy, ethics, social structures, and technology (Ibnu, 2022; Irmayani et al., 2024). Lessons from Gayo Coffee show that institutional inefficiencies, inadequate farmer resources, and misaligned stakeholder visions remain significant challenges (Jaya et al., 2024). Policy reforms should improve institutional clarity, coordination, and integration with community-driven and global sustainability frameworks (Arifin, 2010; Jaya et al., 2024).

Interpretive Structural Modeling (ISM) offers a system-thinking approach to tackle these structural barriers. ISM has been successfully applied in North Toraja and other Indonesian coffee contexts to map stakeholder roles, assess institutional relationships, and design targeted interventions (Fadhil et al., 2018; Prasetyaningtyas et al., 2019; Rico et al., 2024; Sianipar, 2012). It helps to identify leverage points and formulate strategies that strengthen human resources, institutional performance, and cooperative networks. The ISM supports long-term sustainability in complex agricultural systems by linking actors, constraints, and solutions within an integrated framework.

### 4.3 Aligning strategic priorities with systemic barriers

Achieving sustainability in North Toraja's Arabica coffee sector requires aligning strategic priorities with the systemic barriers that hinder progress. Key challenges, such as aging plantations, limited youth participation, climate-related yield fluctuations, and weak farmer bargaining power, are compounded by fragmented institutional support and governance structures (Barreto et al., 2023; Haslinda et al., 2024). These interconnected issues cannot be addressed through one-size-fits-all solutions but require tailored interventions that target leverage points. Using interpretive structural Modeling (ISM), The study identifies a hierarchy of constraints and stakeholder influence to inform systemic actionable strategies (Sia et al., 2025).

One priority is the guaranteed minimum price policy (A1), which addresses the weak farmer bargaining power by reducing price volatility and enhancing income stability. Cooperatives (A2) and local governments (A8) are key to operationalizing this mechanism (Dietz et al., 2018; Rosiana and Feryanto, 2022). Similarly, seed certification programs (A2) directly target productivity losses from aging trees (A3) and poor seed quality (A9), which are essential for long-term agronomic sustainability (Teferi, 2019). Multi-stakeholder platforms (MSPs) (A8) are also vital, fostering dialogue, transparent decision-making, and coordination among farmers, cooperatives, policymakers, and processors (Sia et al., 2025); despite existing limitations, such as weak private sector engagement and limited vertical integration, MSPs have shown success in promoting innovation and collaboration in smallholder systems (Alemu et al., 2021; Barzola Iza et al., 2020; Omore et al., 2015; Thiele et al., 2011).

Digital marketing innovation (A6) complements these efforts by connecting farmers to high-value markets and enhancing competitiveness through improved literacy and traceability systems (Meter et al., 2023; Rugerinyange and Buyinza, 2023). Bridging the generational gap also demands alignment between youth-oriented programs and structural barriers. The Millennial Coffeepreneur Program (A11) positions youth as innovators; however, effective empowerment must go beyond skills to include market access, social capital, and technological support (Anhar et al., 2021; Dzakiroh et al., 2021; Nainggolan et al., 2020; Subagyo et al., 2021). Post-harvest modernization, such as automatic sorting machines (A12) and standardized drying houses (A13), is critical for addressing inefficiencies that undermine product quality.

Institutional and infrastructure innovations, including PERDA on Sustainable Coffee (A4), collective storage warehouses (A3), and long-term technical assistance (A5), further reinforce these strategies. When paired with local seed rejuvenation programs (A14), they create synergies across the ecological, economic, and institutional dimensions. Integrated models from Brazil, Colombia, and Indonesia demonstrate how such approaches enhance sustainability and strengthen bargaining power with the potential for even greater growth in the future (Bacon, 2013; Ramirez-Gomez et al., 2022; Stofya, 2023).

Collectively, these interventions represent a systemic response that redistributes power, reconnects fragmented institutions, and aligns strategies with constraints. By linking root causes to context-specific solutions, North Toraja's experience offers a blueprint for advancing sustainability in smallholder agribusiness systems.

### 4.4 Integrating top-down and bottom-up approaches for sustainable transformation

Sustainable transformation in North Toraja's Arabica coffee sector requires the combination of top-down policies with bottom-up initiatives. While government programs such as seed certification and PERDA (Regional Regulation) on Sustainable Coffee provide the necessary institutional support (Haslinda et al., 2024), their success depends on involving smallholder farmers who hold essential agroecological knowledge but are often excluded from decision-making (Rosiana and Feryanto, 2022). Studies show that relying only on value chain interventions such as certification is insufficient without farmer participation and local relevance (Neilson and Shonk, 2014). The ISM results of The study confirm that connecting institutional support with farmer-driven actions such as regeneration programs and multi-stakeholder platformsis key to creating meaningful and lasting sustainability (Samper and Quiñones-Ruiz, 2017).

Bottom-up mechanisms such as cooperatives, local agroforestry practices, and community innovation platforms—ground policy frameworks in the lived realities of farmers, support co-designed solutions, and build local governance capacity (Anhar et al., 2021; Sia et al., 2025). Cooperatives (A2) play an intermediary role by connecting farmers to markets, certification, and policy channels, although their effectiveness is often constrained by underfunding and weak institutional support. To unlock their potential, governments and development actors must move beyond outreach toward participatory strategies that center on collaboration and listening. Research shows that reducing transaction costs, investing in producer organizations, and leveraging digital tools, such as (specific examples of digital tools), enhances farmers' market access and agency (Borrella et al., 2015; Panggabean et al., 2022; Pingali et al., 2019).

Inclusive platforms that integrate top-down and bottom-up strategies are essential to aligning stakeholder interests. ISM analysis highlights that platforms supported by government agencies (A8), companies (A1), and cooperatives can coordinate actors, bridge governance levels, and generate shared values (Dietz et al., 2018; Sia et al., 2025). Such platforms ensure context-sensitive and socially embedded sustainability planning, whereas ISM and Smart ISM offer tools to identify interlinkages and guide strategy development (Ahmad and Qahmash, 2021; Butler et al., 2015; Sianipar, 2012).

A systems thinking approach reinforces the need for feedback loops between policymaking and grassroots innovation. Participatory monitoring, farmer-led evaluations, and adaptive learning strengthen the responsiveness of top-down interventions and increase the relevance of local innovations (Contreras-Medina et al., 2024; Harvey et al., 2021). Smallholder systems operate as complex adaptive systems that require flexible and dynamic governance structures supported by socio-ecological frameworks (Amandaria et al., 2025; Orr et al., 2018; Rich et al., 2015).

Sustainable transformation requires a hybrid governance model that blends formal institutions with local agencies, and fosters trust-based collaboration across scales. This model encourages shared responsibility among farmers, governments, and civil society (Meter et al., 2023; Panggabean et al., 2022), thus complementing buyer-driven sustainability governance with inclusive community mechanisms (Grabs, 2017). Relational governance, built on cooperation and joint problem solving, has effectively addressed complex challenges such as climate change and crises such as COVID-19 (Akbar et al., 2025). The Mediated Partnership Model from Indonesia exemplifies how bottom-up efforts can align with global standards while prioritizing farmers' needs (Wijaya et al., 2017).

### 4.5 Implications for policy, institutional coordination, and future research

The study presents key policy implications to enhance sustainability in the North Toraja Arabica coffee sector. This underscores the need for policies that balance market orientation with social protection, such as guaranteed minimum price schemes and targeted input subsidies for smallholders (Meter et al., 2023). Adaptive policies should support these efforts by responding to climate variability and demographic changes (Contreras-Medina et al., 2024). It is crucial that all stakeholders, including local governments, farmers, and policymakers, collaborate to institutionalize sustainable coffee development through region-specific legal instruments—such as the proposed PERDA on Sustainable Coffee—to ensure long-term support for infrastructure, farmer assistance and climate-resilient practices (Haslinda et al., 2024).

Future studies should explore these three key areas. First, deeper empirical studies are needed to assess the micro-level effects of sustainability strategies such as seed certification, mechanized post-harvest tools, and digital marketing on household income, gender equity, and labor dynamics. Second, investigating the political economy of coffee governance will help unpack how power asymmetries among traders, processors, and farmers influence access to markets, certifications, and climate finance (Harvey et al., 2021; Rosiana and Feryanto, 2022). Third, comparative studies across other specialty coffee-producing regions in Indonesia and Southeast Asia can help to identify context-specific and transferable best practices.

The study design is qualitative and based on key informants; therefore, the findings are intended for analytical generalization (understanding the role-constraint-coordination mechanism), not for statistical estimation of the population. Data credibility was strengthened through triangulation between stakeholder categories, checking the consistency of findings across sources, and selecting informants until the information obtained showed a recurring trend (information redundancy). Limitations remain, particularly the potential for perspective bias if some groups are underrepresented. We have included this as a limitation and an agenda for further research, such as quantitative survey design or broader focus group discussions.

Interpretive structural Modeling (ISM) has proven to be an effective tool for analyzing and improving coffee agribusiness systems in Indonesia. Applications of ISM include actor mapping in North Toraja (Rico et al., 2024), development of profit-sharing frameworks (Sianipar, 2012), strategies for Robusta coffee rejuvenation (Nuddin et al., 2019), and institutional development in the Gayo agroindustry (Surur et al., 2014). These studies demonstrate ISM's value of the ISM in diagnosing system dynamics, identifying leverage points, and guiding strategic interventions (Fadhil et al., 2018; Nuddin et al., 2019; Rico et al., 2024; Sianipar, 2012). A limitation is that intermediaries were not included as direct interview participants, which may under-represent their rationales and constraints. While we mitigated this through triangulation across other stakeholder groups and documentary/observational evidence, future research should incorporate intermediaries to further validate and refine the actor-relationship structure.

## 5 Conclusion and policy implications

The sustainability of North Toraja's Arabica coffee sector is shaped by complex institutional arrangements, power relations, and sociocultural norms that influence access to resources, decision-making, and market participation. Findings from systems thinking and Interpretive Structural Modeling (ISM) analysis show that upstream institutional reforms—such as guaranteed minimum prices, certified seed systems, and stronger farmer organizations—are critical leverage points that can drive equitable value chain transformation. Downstream opportunities, including youth engagement, post-harvest innovation, and digital marketing, depend on these fundamental changes. Achieving inclusive transformation requires bridging top-down governance with bottom-up empowerment through multistakeholder platforms, participatory decision-making, and context-sensitive institutional design.

To clarify the proposed multistakeholder platforms, this study refers to a locally anchored coordination mechanism (e.g., a district-level coffee forum/coordination council) convened by the Agriculture Office and involving farmer groups/cooperatives, extension services, processors/MSMEs, and private-sector buyers. Such a platform can function to (i) coordinate shared priorities and sequencing of interventions (e.g., seed certification, farm rejuvenation, and extension support), (ii) improve transparency around quality and pricing requirements, (iii) align investments in shared infrastructure (e.g., storage and drying facilities), and (iv) support joint monitoring of sustainability commitments, including the proposed PERDA on Sustainable Coffee.

By “context-sensitive institutional design,” we mean tailoring rules, incentives, and governance arrangements to North Toraja's sociocultural and institutional realities—such as traditional authority structures, local norms, and the existing capacities of cooperatives and public agencies—so that reforms are legitimate, feasible, and adopted over time. Policy priorities should therefore emphasize long-term institutional strengthening, capacity building for cooperatives, and fostering trust and collaboration across value chains. Interventions must also account for the embedded nature of coffee production within traditional authority systems, evolving community aspirations, and competing visions for rural development. Aligning institutional reforms with inclusive governance and social empowerment can enhance resilience, equity, and competitiveness, offering lessons for smallholder-dominated commodity systems in the Global South.

## Data Availability

The raw data supporting the conclusions of this article will be made available by the authors, without undue reservation.
