# Supplementary material for: Institutional dynamics, stakeholder interactions, and sustainability pathways in North Toraja's Arabica coffee sector: a systems approach to inclusive agribusiness transformation
Source: Front Sociol. 2026 Mar 12;11:1684951. doi: 10.3389/fsoc.2026.1684951 (PMC13017354; doi:10.3389/fsoc.2026.1684951)
Supplement: Supplementary file 1 [file Supplementary_file_1.docx]

**Supplementary Materials**

**TABLE S1**.

Number of Research Informants as Arabica Coffee Agribusiness Actors.

| **No.** | **Arabica Coffee Agribusiness Players** | **Number of Informants** |
| --- | --- | --- |
| 1 | Agriculture Office of North Toraja District | 2 |
| 2 | Coffee Company | 3 |
| 3 | Cooperative | 3 |
| 4 | Farmer Group | 5 |
| 5 | Farmers | 5 |
| 6 | Coffeeshop | 3 |
| 7 | MSME | 3 |
| **Total** | | **17** |

**TABLE S2.**

Elements and Sub-Elements of Actors, Constraints, and Strategies for Arabica Coffee Sustainability in North Toraja District.

| **Elements** | **Sub-Element** |
| --- | --- |
| Institutions that Play a Role in the Sustainability of Arabuka Coffee in North Toraja District | 1. Company (A1) 2. Cooperative (A2) 3. Coffeeshop (A3) 4. MSME (A4) 5. Local Trader (A5) 6. Agricultural Extension Worker (A6) 7. Agricultural Machinery Industry A7) 8. Agriculture Office (A8) 9. Chemical Input Industry (A9) 10. Farmer Group (A10) 11. Farmer (A11) |
| Constraints to Coffee Agribusiness Sustainability in North Toraja District | Domination of elderly coffee farmers (A1)  Farmer group coaching is not optimal (A2)  Coffee plants that have passed the optimal productive period (>20 years) (A3)  Coffee production variability due to climate change impacts (A4)  Low interest in coffee farmer regeneration (A5)  Traditional post-harvest technology (A6)  Limited access to financing (A7)  Weak bargaining position of farmers in coffee price determination (A8)  Seedling growth failure (A9)  Limited APBD budget for coffee assistance (A10)  Digital marketing of farmers is not yet developed (A11)  Inter-regional coffee bean blending practices (A12) |
| Sustainability Strategy of Coffee Agribusiness in North Toraja District | Basic coffee price policy secured (A1)  Arabica coffee plant seed certification (A2)  Construction of collective coffee storage warehouse (A3)  Drafting of PERDA on Sustainable Coffee (A4)  Sustainable technical assistance program (5 years) that includes cultivation, post-harvest, and marketing training (A5)  Coffee Digital Marketing Innovation (A6)  Sustainable export partnerships (A7)  Establishment of multi-stakeholder platform (A8)  Integrated harvest transportation services (A9)  Development of Agro-tourism-based Coffee Village (A10)  Millennial coffee farmer education program (Young Coffeepreneur) (A11)  Adoption of an automatic coffee sorting machine (A12)  Provision of standardized coffee drying houses (A13)  Coffee rejuvenation program based on superior local seeds (A14) |

**TABLE S3.** Final Canonical Matrix (Tabular View)

| **Element ei** | **FRMi1** | **...** | **FRMin** | **Driver Power Di** | | **Dependence Ri** | **Rank/Level** | |
| --- | --- | --- | --- | --- | --- | --- | --- | --- |
| e1 | 0 or 1 | ... | 0 or 1 | Count of 1s in row i | | Count of 1s in column | Hierarchical | |
| ... |  |  |  |  | |  |  | |
| En |  |  |  |  | |  |  | |
| En |  |  |  |  | |  |  | |
| Level 1  Level 2  Level 3  Level 4  A5  Level 5  A3  A8  A10  A11  A4  A6  A1  A2  Level 6  A7  A9 | | | | | | | |  |
|  | | | | | | | |  |
| Description:   1. Company (A1) 2. Cooperative (A2) 3. Coffeeshop (A3) 4. MSME (A4) 5. Local Trader (A5) | | | | | 1. Local Trader (A5) 2. Agricultural Extension Worker (A6) 3. Agricultural Machinery Industry (A7) 4. Agriculture Office (A8) 5. Chemical Input Industry (A9) 6. Farmer Group (A10) 7. Farmer (A11) | | |  |
| **FIGURE S1.**  Structure of the Actors in the Arabica Coffee Agribusiness's Sustainability in North Toraja District | | | | | | | |  |

**Level 1**

**Level 2**

**Level 3**

**Level 4**

**Level 5**

C1

C3

C5

C8

C2

C9

C6

C7

**Level 6**

C10

C4

C11

C12

|  | |
| --- | --- |
| Description   - - - 1. Domination of elderly coffee farmers (C1)       2. Farmer group coaching is not optimal (C2)       3. Coffee plants that have passed the optimal productive period (>20 years) (C3)       4. Coffee production variability due to climate change impacts (C4)       5. Low interest in coffee farmer regeneration (C5)       6. Traditional post-harvest technology (C6) | - - - 1. Limited access to financing (C7)       2. Weak bargaining position of farmers in coffee price determination (C8)       3. Seedling growth failure (C9)       4. Limited APBD budget for coffee assistance (C10)       5. Digital marketing of farmers is not yet developed (C11)       6. Inter-regional coffee bean blending practices (C12) |
| **FIGURE S2.**  Structure of Constraints that Impede Arabica Coffee's Sustainability | |

| Level 1  Level 2  Level 3  Level 4  S3  Level 5  S7  S5  S1  S2  S6  S9  S4  S10  Level 6  S8  S11  S12  S13  S14 | |
| --- | --- |
| **Description:**   1. **Basic coffee price policy secured (S1)** 2. **Arabica coffee plant seed certification (S2)** 3. **Construction of collective coffee storage warehouse (S3)** 4. **Drafting of PERDA on Sustainable Coffee (S4)** 5. **Sustainable technical assistance program for 5 years (cultivation, post-harvest, and marketing) (S5)** 6. **Coffee Digital Marketing Innovation (S6)** 7. **Sustainable export partnerships (S7)** | 1. **Establishment of multistakeholder platform (S8)** 2. **Integrated harvest transportation services (S9)** 3. **Development of Agro-tourism-based Coffee Village (S10)** 4. **Millennial coffee farmer education program (Young Coffeepreneur) (S11)** 5. **Adoption of an automatic coffee sorting machine (S12)** 6. **Provision of standardised coffee drying houses (S13)** 7. **Coffee rejuvenation program based on superior local seeds (S14)** |
| **FIGURE S3.**  Strategic Program Structure for Arabica Coffee Susin North Toraja | |
